# Successful use of montelukast in eosinophilic gastroenteritis: a case report and a literature review

**DOI:** 10.1186/s12876-021-01854-x

**Published:** 2021-07-08

**Authors:** Emran A. El-Alali, Ibrahim M. Abukhiran, Tarik Z. Alhmoud

**Affiliations:** 1 Department of Internal Medicine, Anne Arundel Medical Center, 2001 Medical Pkwy, Annapolis, MD 21401 USA; 2 Anatomic and Clinical Pathology, University of Iowa Hospitals and Clinics, 200 Hawkins Dr, Iowa City, Iowa, USA; 3 Promedica Digestive Health Care, 5700 Monroe St, Suite 103, Sylvania, OH USA

**Keywords:** Montelukast, Eosinophilic gastroenteritis, Leukotriene antagonist, Case report

## Abstract

**Background:**

Eosinophilic gastrointestinal disorders, also known as eosinophilic gastroenteritis, are rare inflammatory conditions characterized by eosinophilic infiltration of different parts of the gastrointestinal tract, along with peripheral eosinophilia in most cases. Other known causes for gut eosinophilic infiltration must be excluded to confirm the diagnosis of eosinophilic gastroenteritis. Symptoms of the disorder depend on the affected gastrointestinal tract segment and depth of involvement. Treatment includes systemic glucocorticoids and/or dietary therapy with an empiric elimination diet. Second line therapies include the leukotriene receptor antagonist montelukast, and other anti-allergy drugs such as mast cell stabilizers (including cromolyn and the H1-antihistamine ketotifen), suplatast tosilate which is a selective Th-2 cytokines (IL-4 and IL-5) inhibitor, and the monoclonal anti-IgE antibody omalizumab. We report a case of eosinophilic gastroenteritis who was successfully treated and achieved remission with montelukast as an initial monotherapy. Upon extensive literature review, this represents the second reported adult case of eosinophilic gastroenteritis who responds to montelukast alone as a first line therapy.

**Case presentation:**

A 49-year-old female presented with recurrent abdominal pain, vomiting, diarrhea and unexplained eosinophilia. She was diagnosed with eosinophilic gastroenteritis and was successfully treated with montelukast monotherapy. After 7 days of therapy, the patient responded well and had complete resolution of her gastrointestinal symptoms and peripheral eosinophilia. Patient remained in remission on follow-up after 12 months. We reviewed the literature for leukotriene antagonist use in the treatment of eosinophilic gastroenteritis and included the cases treated with the leukotriene antagonist montelukast as an initial therapy or as a second line therapy for refractory disease.

**Conclusion:**

Montelukast may be an effective treatment for eosinophilic gastroenteritis, either alone or in combination with systemic steroids or ketotifen. Our patient is the second reported adult case of eosinophilic gastroenteritis who responded to montelukast alone as a first line therapy. Further studies and clinical trials are required to confirm efficacy compared to standard therapy.

## Background

Eosinophilic gastroenteritis (EGE) is a rare inflammatory condition that can affect patients of any age and is characterized by eosinophilic infiltration of different parts of the gastrointestinal tract. Stomach and/or duodenum are most commonly affected. Tissue and peripheral Eosinophilia are usually present. The inflammation occurs without any other underlying cause of eosinophilia. The estimated prevalence rate of EGE is 1.7–8.4 per 100,000 [1–3]. The rate of diagnosis has increased overtime [4].

Etiology is unclear, but an allergic component is highly suggested based on epidemiological and clinical features [5]. Although the role of food allergy in EGE has not been as clearly defined as with eosinophilic esophagitis, several reports have described an improvement in disease activity with an elimination diet [6].

Symptoms vary based on the location, extent and depth of involvement of the gastrointestinal tract. The most common symptoms of eosinophilic mucosal infiltration are abdominal pain, nausea, early satiety, vomiting, diarrhea and weight loss. Involvement of the muscular layer results in wall thickening and affects mobility, so patients may present with symptoms of intestinal obstruction. Patients with subserosal type of EGE usually present with ascites, either isolated or with symptoms of mucosal or muscular EGE.

The diagnosis of EGE is based on the presence of eosinophilic infiltration of the gastrointestinal (GI) tract on biopsy, lack of involvement of other organs and absence of other causes of intestinal eosinophilia by history, laboratory evaluation and other testing.

First line therapy is empiric elimination diet and/or systemic glucocorticoids depending on disease severity [7].

We report a case of a middle-age woman with EGE who responded to montelukast (MK), a leukotriene antagonist, as a first line treatment. She did not follow diet therapy instructions and refused systemic steroids. All symptoms of gastroenteritis and colitis including abdominal pain, vomiting and diarrhea, and peripheral eosinophilia resolved and she remained symptom free on a 12-month follow up.

The existing literature on the use of MK in EGE revealed one reported adult case who responded to MK as a first line monotherapy. Our literature search also revealed several cases of EGE who responded to MK when combined with other drugs or to MK as a second line therapy.

## Case presentation

A 49-year-old Caucasian woman, with past medical history of diabetes mellitus type II, dyslipidemia and hypertension, presented to the emergency department with cramping-like abdominal pain, vomiting and diarrhea that started few weeks prior to presentation. Abdominal pain was generalized before becoming localized to the epigastrium, without radiation. No alleviating or aggravating factors were noted. She had frequent, watery to loose, non-bloody bowel movements.

Patient denied dysphagia or odynophagia, and had no fever, chills, skin rashes or joint pains. She denied any recent travel, sick contact or antibiotics use prior to onset of symptoms. Review of systems was unremarkable. Surgical history included cholecystectomy. Patient did not use tobacco, alcohol or illicit drugs. No family history of similar symptoms.

Patient was admitted to the hospital for further management. Prior to hospitalization, patient was evaluated and treated at two different healthcare facilities (including one hospital admission) for similar symptoms. There, she had a computed tomography (CT) scan of the abdomen and pelvis with contrast that showed fluid-filled bowels and thickened colonic wall, and she was released from the hospital on empirical antibiotics for presumed infectious enterocolitis, but symptoms recurred soon after hospital discharge.

On physical examination the patient appeared in moderate pain and distress. Vital signs showed an elevated blood pressure of 146/93 mmHg, heart rate of 88 beats per minute and a normal temperature of 36.5 °C.

Abdominal exam showed generalized tenderness that was worse in the epigastrium with no rebound tenderness or abdominal distention. Bowel sounds were normal. There was no palpable organomegaly.

Eyelids showed xanthelasmas. Physical examination of heart, chest and extremities was normal.

Lab workup revealed peripheral eosinophilia; total white blood cells were 9,000 cells/µL with 28% eosinophils (absolute eosinophil count of 2520 eosinophils/µL). Hemoglobin level was normal (14.7 g/dL).

Lipase was 2831 IU/L (compared to 2415 IU/L 5 days earlier on patient’s recent hospitalization). Alkaline phosphatase was 391 IU/L, with liver transaminases AST and ALT slightly higher than the normal range (57 U/L and 54 U/L, respectively) and a normal bilirubin level. Kidney function, lactic acid and electrolytes were within normal limits.

Erythrocyte sedimentation rate and C-reactive protein were within normal ranges (10 mm/h and 0.673 mg/dL, respectively).

Evaluation for eosinophilia included a peripheral blood smear that showed an increased number of mature-looking eosinophils and no evidence of atypical cells or blasts. HIV test was negative. IgG-4 level was 40 mg/dL (normal < 86 mg/dL). Chest X-ray showed no infiltrates or hilar adenopathy. Stool pathogen PCR panel was negative for Shigella, *E. coli*, Campylobacter, Clostridioides difficile toxin A/B, Salmonella, cryptosporidium and cyclospora and enteric viruses. Microscopic testing for stool ova and parasites was negative. All patient’s medications were reviewed, and the possibility of drug-induced eosinophilia was excluded.

CT scan of the abdomen and pelvis showed diffuse fluid-filled loops of small and large bowel without bowel obstruction. Normal appearing pancreas with no evidence of inflammation or pseudocysts. No biliary dilatation and surgically absent gallbladder due to prior cholecystectomy. The liver and spleen were not enlarged and there was no lymph nodes enlargement.

**Fig. 1:**
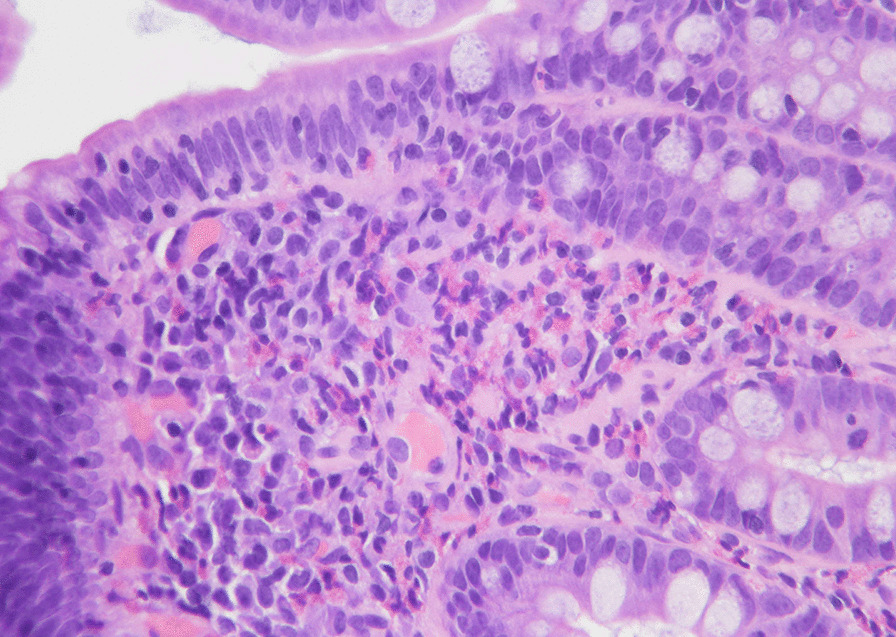
Duodenum biopsy showing markedly increased eosinophils (> 20/hpf) in lamina propria with focal epithelial involvement. (Hematoxylin and Eosin 40x)

A consultation for GI service was obtained, and because of the persistent unexplained symptoms and eosinophilia the patient underwent esophagogastroduodenoscopy (EGD), mucosal biopsies were obtained from the esophagus, stomach, duodenum and jejunum. Histological exam revealed eosinophilic-predominant mucosal inflammatory infiltrates in all biopsied specimens as shown in Fig. 1. Helicobacter Pylori was negative. No villous abnormality, cryptitis or crypt abscesses were identified. Diagnosis of eosinophilic gastroenteritis (EGE) and eosinophilic esophagitis was made.

Patient was initially managed with supportive therapy including nothing by mouth (NPO), intravenous (IV) fluid infusion and analgesia. No antibiotics were given during the last hospital admission. Patient refused to start therapy with steroids due to concerns about adverse effects. She was started on montelukast 10 mg once a day by mouth and discharged home to follow as an outpatient. Abdominal pain, vomiting and diarrhea resolved over 1 week of treatment.

Clinic follow-up was made 5 months and 12 month post hospitalization. The patient reported no further symptoms and reported adherence to montelukast therapy without any adverse effects. Complete blood count (CBC) was repeated with findings of normal eosinophils [absolute eosinophil count of 170 eosinophils/µL].

## Discussion

This is a case of a 49-year-old woman who suffered from multiple gastrointestinal symptoms including nausea, vomiting, abdominal pain and diarrhea, which are commonly encountered in the healthcare setting. She presented to the emergency department few times and was hospitalized without identifying the underlying diagnosis, and had no relief of her symptoms. We were involved in the care of this patient on the second hospital admission, and based on the constellation of symptoms, laboratory results and CT scan findings we came to an initial differential diagnosis of infectious gastroenteritis or parasitic infection. Acute pancreatitis with ileus was a concern due to markedly elevated lipase, however, the absence of typical pancreatitis-related epigastric pain and the normal appearance of pancreas on two CT scans ruled out pancreatitis as a cause for her symptoms. Stool microbiology studies excluded viral, bacterial or parasitic infections. Patient did not respond to standard therapies including antibiotics (at the initial hospital admission), bowel rest with NPO, analgesia and IV fluids administration.

Given that the patient had persistent unexplained peripheral eosinophilia, bowel wall thickening on CT scan and negative workup for common disorders, along with the non-resolving nature of symptoms despite standard treatment, a diagnosis of eosinophilic gastrointestinal disorder was presumed. Upper endoscopy with biopsies confirmed the diagnosis of EGE (mucosal type), based on histopathologic findings of abundant eosinophils in the gastric, duodenal and jejunal mucosa. Montelukast (MK) was then started based on patient’s preferences and concerns about corticosteroids adverse effects. She responded well and became asymptomatic over 1 week of therapy, and peripheral eosinophil count has normalized. Patient remained in remission during follow-up 12 months later.

EGE is a rare disease, therefore evidence for treatment is based on limited case reports and clinicians' experience [8]. MK is a safe medicine and has a low side effect profile compared to systemic steroids [9]. MK is a selective and competitive leukotriene receptor inhibitor. Cysteinyl leukotrienes have potent chemoattractant properties for eosinophils. Together with interleukin 3 and 5, cysteinyl leukotrienes play a major role in the recruitment of eosinophils into the affected tissue leading to tissue damage [10].

## Literature review

We reviewed the literature on montelukast (MK) use in EGE cases and summarized the reported cases in Tables 1, 2 and 3. MEDLINE, Embase and Google scholar databases were searched for case reports, case series and clinical trials using the following terms: “eosinophilic gastroenteritis”, “eosinophilic gastritis”, eosinophilic duodenitis”, “eosinophilic colitis” AND “montelukast” OR “leukotriene antagonist”. Studies that confirmed the diagnosis of EGE using the following criteria were included: Symptoms of EGE including abdominal pain, nausea, vomiting, diarrhea, weight loss, failure to thrive or iron deficiency anemia; eosinophilic infiltrate ≥ 20/hpf; and absence of other causes of eosinophilia or eosinophilic infiltrate. Studies that used a lower cutoff for eosinophilic infiltrate (i.e., < 20/hpf) or used the diagnosis of “functional dyspepsia with eosinophilia or duodenal eosinophilia” were excluded. Cases of eosinophilic esophagitis were also excluded.

**Table 1:** Montelukast (MK) as a first line monotherapy in eosinophilic gastroenteritis (EGE)

Authors	Study design	Number of patients, gender (M%), mean age ± SD (years)	Affected segments	Dose, duration	Outcomes: EGE symptoms, tissue eosinophilia (if reported)	Follow-up duration
*Adult cases*						
Wong et al. [11]	Retrospective case series^a^	One M, –	–	10 mg daily, 1 month	Symptoms improved, –	10 months
*Pediatric cases*						
Tien et al. [12]	Retrospective case series^a^	4, M 75%, 8 ± 4.3^b^	Stomach and duodenum	5 mg daily, –	Symptoms resolved in 4/4 cases, Eosinophilia improved 3/3 cases	12 months
Selva Kumar et al. [13]	Case report	One F, 12	Stomach and duodenum	10 mg daily, 1 month	Symptoms and eosinophilia resolved	–
Neustrom and Friesen [14]9	Case report	One F, 13	Esophagus, stomach and duodenum	10 mg daily, –	Symptoms resolved, Eosinophilia decreased	4 months

*M* male, *F* female, *mg* milligrams, *SD* standard deviation

^a^Asian population

^b^Mean age ± SD for all study population

**Table 2:** Use of montelukast (MK) as part of an initial combination therapy in eosinophilic gastroenteritis (EGE)

Authors	Study design	Number of patients, gender (M%), mean age ± SD (years)	Affected segments	Medications used, duration (if known)	Outcomes: EGE symptoms, tissue eosinophilia and follow-up duration (if reported)
Priyadarshni et al. [16]	Case report	One M, 28	Stomach and small bowel	Prednisone and montelukast (dose/duration: not reported)	Symptoms resolved
Hui and Hui [15]	Prospective	64, M 63%, 40.5 ± 23.5^b^	Terminal ileum and/or colon	Ketotifen 1 mg daily up to 2 mg twice daily and montelukast 10 mg daily, 16 weeks	Symptoms resolved in 57 of 64 (89.1%)
Chen et al. [17]	Care report	One M, 54	Stomach and duodenum	Methylprednisolone 30 mg daily and montelukast 4 mg daily, 1 month	Symptoms resolved, Eosinophilia improved
Wong et al. [11]	Retrospective case series^a^	One M, –	–	Prednisolone 30–40 mg/day tapered over 1–3 months and montelukast (MK dose/duration: unknown)	Symptoms and eosinophilia resolved, 36 months
Baek et al. [18]	Case report	One F, 68	Duodenum	Prednisolone 30 mg daily, 1 month Montelukast 10 mg daily, 5 months	Symptoms and eosinophilia resolved
Milić et al. [19]	Case report	One F, 30	Esophagus, stomach, small and large intestine	Prednisone 40 mg daily Montelukast 10 mg daily, 2 weeks	Symptoms resolved
*Pediatric cases*					
Tien et al. [12]	Retrospective case series^a^	8, M 63%, 7.9 ± 6.5^b^	Stomach and duodenum	Corticosteroids (CS) 1–2 mg/kg/day CS and MK in 6/8 CS and MK and Ketotifen in 2/8, Duration: unknown	1/8 (12.5%) in remission (on CS and MK)

*M* male, *F* female, *mg* milligrams, *MK* montelukast, *SD* standard deviation

^a^Asian population

^b^Mean age ± SD for all study population.

**Table 3:** Reported use of Montelukast (MK) as a subsequent or adjunctive therapy in eosinophilic gastroenteritis (EGE)

Author(s)	Study design	Number of patients, gender (M%), mean age ± SD (years)	Affected segments	Medication(s) prior to starting MK, MK dose and duration (if known)	Outcomes	Follow up duration
*Adult cases*						
Schwartz et al. [24]	Case report	One M, 27	Small intestine	Prednisone 20 mg daily (induction for 4 weeks), Montelukast 10 mg daily, 20 months	Symptoms resolved	20 months
Muller et al. [22]	Prospective	Two, – 39.5 ± 19.5^b^	Stomach and small intestine	Prednisolone 40 m daily, Montelukast (dose/duration: not reported)	Symptoms resolved	14 ± 5.6 months
De Maeyer et al. [21]	Case report	One M, 38	Duodenum	Methylprednisolone 16 mg daily (tapered to 4 mg) Montelukast 10 mg daily	Symptoms improved	Unknown
Urek et al. [25]	Case report	One M, 18	Serosal disease	Prednisolone 20 mg daily Montelukast 10 mg daily	Remission after 4 weeks of montelukast	Unknown
*Pediatric cases*						
Lu and Ballas [23]	Retrospective	Two, M 50%, 17 and 39	–	Prednisone 26–40 mg QOD Prednisone 30 mg daily	MK decreased prednisone requirement to 10 mg QOD in pediatric case	Unknown
Quack et al. [20]	Case report	One F, 17	Esophagus, stomach, ileum and colon	Prednisone 40 mg daily (tapered to 10 mg: relapsed) Montelukast 10 mg daily	Symptoms resolved	24 months
Menon et al. [26]	Case report	One F, 11	Stomach and small bowel	Prednisolone (P): Relapsed after discontinuation Montelukast: Added to 5 mg P	Symptoms resolved	Unknown
Vithayasai et al. [27]	Retrospective^a^	One, – 6.75 ± 6.25^b^	–	Prednisolone (failed) Montelukast and ketotifen were started	No relapses	3 months to 5 years

*QOD* every other day, *F* female, *M* male, *mg* milligrams, *MK* montelukast, *SD* standard deviation

^a^Asian population

^b^Mean age ± SD for all study population

Our patient is the second adult case of EGE in the literature who successfully responds to initial MK monotherapy, and the first reported adult Caucasian patient. Wong et al. reported one adult case and six pediatric cases in an Asian population. Our patient remained on MK for 1 year, during which she maintained remission, whereas the adult patient reported by Wong et al. used MK for 1 month and remained in remission after 12 months. In one systematic review, EGE was found to occur in Asians more than Caucasians, while eosinophilic esophagitis had an opposite trend [28]. Table 1 describes the reported use of MK as a first line monotherapy in a total of seven EGE cases.

Table 2 shows the use of MK in EGE as an initial combination therapy with systemic glucocorticoids in a total of 13 patients (including 5 adults); 46% responded well and had symptoms resolution (100% of the adult cases) while on glucocorticoid and MK combination therapy. In a large case series of 64 patients, MK was combined with the mast cell stabilizer and H1 antagonist ketotifen; 89% responded well to this combination with resolution of symptoms [16].

Table 3 describes the successful use of MK as a steroid-sparing agent in a total of eight patients (63% adults) [20–26], or as a second line therapy after failing corticosteroids in one child with EGE [27].

The merits of our approach for induction of remission using MK is that MK is a safe medication with a lower side effects profile compared to systemic corticosteroids. The limitations to our approach is the absence of large prospective studies to prove and support its effectiveness, duration and long term use for this purpose in EGE.

Based on data from our patient and from similar case reports, we suggest the use of MK for at least one month to induce remission in EGE

## Conclusion

Montelukast (MK) might be an effective treatment for EGE when used as an initial therapy alone, in combination with systemic steroids or ketotifen, or as a steroid-sparing agent. Our patient is the second reported adult case of EGE with successful remission following treatment with MK alone. The majority of reports in the literature concerning MK use in EGE have shown positive and promising outcomes. Larger prospective series and clinical trials are needed to further delineate the use of MK treatment in EGE, to include the exact dosage, duration and long-term outcomes.

## Data Availability

The datasets used and/or analysed during the current study are available from the corresponding author on reasonable request.

## Abbreviations

EGE: Eosinophilic gastroenteritis
MK: Montelukast
GI: Gastrointestinal
CT: Computed tomography
NPO: Nil per os (nothing by mouth)
IV: Intravenous
EGD: Esophagogastroduodenoscopy
